# Regulations and practices of structured doctoral education in the life sciences in Germany

**DOI:** 10.1371/journal.pone.0233415

**Published:** 2020-07-30

**Authors:** Daniel Lachmann, Thilo Martius, Julia Eberle, Mareike Landmann, Lena von Kotzebue, Birgit Neuhaus, Stefan Herzig

**Affiliations:** 1 Vice-Rectorate for Studies and Teaching, University of Cologne, Cologne, Germany; 2 Educational Psychology, Ruhr-Universität Bochum, Bochum, Germany; 3 University of Cologne, Cologne, Germany; 4 Didaktik der Bio- und Geowissenschaften, School of Education, Universität Salzburg, Salzburg, Austria; 5 Institute for Biology Education, Ludwig-Maximilians-Universität München, Munich, Germany; 6 TH Köln (University of Applied Sciences) of Cologne, Cologne, Germany; Florida Agricultural and Mechanical University, UNITED STATES

## Abstract

Structured doctoral education is increasingly preferred compared to the individual model. Several science policy organisations give recommendations on how to structure doctoral education. However, there is little research on to what extent these recommendations find their way into practice. In our study, we first compared European and German recommendations on doctoral education with, second, the institutional regulations of structured doctoral programmes (*N* = 98) in the life sciences at twelve different German universities. Additionally, we third asked doctoral graduates (*N* = 1796) of these structured doctoral programmes and graduates of individual doctoral studies about their experience in doctoral education. Fourth, we contrasted the regulations of structured doctoral programmes with the reported experiences of their graduates. We found significant deviations of the reported practices of graduates from the regulations of their organisations, regarding the student admission, supervision and curricular activities of doctoral candidates. The efficacy of structured versus traditional doctoral education should be examined based on reported practice rather than on the respective written regulations.

## 1 Introduction and background

Professionalisation of doctoral education, its massification and implementation of quality assurance measures are global trends in doctoral education [1, 2]. In Europe, the Bologna process was a major driver of change in several countries, aiming to focus doctoral training on developing research skills and preparing graduates for the labour market outside academia [3]. In Germany as well as in other European countries, this change is expected to happen by reforming the traditional forms of doctoral training in terms of structuring and restructuring doctoral education [4–6]. Recommendations for such structuring of doctoral education are being implemented by several European countries [7]. However, many European countries still maintain a combination of different approaches to doctoral education [8]. There are two apparently dichotomous approaches to doctoral education—individual and structured. However, upon closer examination, as will be demonstrated in this paper, the distinction between the two approaches appears to be vaguer than the dichotomy suggests. The traditional or individual model is often referred to as the “master-apprentice-model”, characterized by an individual agreement between a doctoral student and a supervisor in which the supervisor guides the individual, curiosity-driven research process of their ‘apprentice’ [4]. In this form of doctoral education, responsibility is shared by the doctoral students and their supervisor, whereas in the structured form, much of the responsibility is transferred to the academic organisation [9]. Structured forms of doctoral education are part of institutional strategies and need to be scalable to an increasing number of doctoral candidates who engage in knowledge production [9], requiring a common structuring frame for doctoral studies including certain steps within the process.

Structured doctoral education is expected to solve problems such as observed inequality in who pursues a doctorate due to non-transparent admission processes and long phases of career insecurity starting with often unnecessarily lengthy duration of doctoral studies [4, 9, 10]. In addition, doctoral education is progressively expected to prepare doctoral students for the labour market outside academia, as most doctoral graduates do not pursue an academic career in the long run [11]. Consequently, various stakeholders have recommended or discussed the implementation of structured doctoral education [12, 13].

To deal with the challenges of doctoral education today, organisations are supposed to implement various political recommendations. These include the implementation of additional supervisors or a supervision committee, the establishment of a mandatory curriculum, creating work groups, improving funding opportunities via scholarships, setting a time frame for the completion of the thesis and clarifying the formal status of the candidates within the respective organisations ([6], for an overview of typical criticisms and recommendations see, e.g., pages [14–17]).

However, there is currently little to no research that investigates if and how organisations implement these recommendations. Therefore, the core question of this paper is whether and to what extent these recommendations have found their way into the practices of doctoral education, using the German doctoral education system as an example.

Since Germany has a comparatively high share of doctoral graduates in general and life scientists are particularly likely to pursue a doctorate, both, the country and this specific discipline may function as excellent cases to elaborate the issue of regulations (of which some are inherently specific for any given nation) and practices of doctoral education. In addition to the high share of doctoral graduates in the life sciences, many authors recommend discipline specific analyses due to often quite different cultures concerning general and doctoral training [18–20]. This puts the life sciences in a predestined position to serve as a case study. Furthermore, many of the recommendations found in the literature come from international institutions and are relevant for several European countries beyond Germany [7, 8]. The results presented in this paper can, therefore, serve for comparison between different European countries or build the base for future discipline specific research regarding the current state of implementation of recommendations into doctoral education.

In our investigation, we first elaborated on how policy suggests remedying the problems of doctoral education. In the next step, we searched for these suggestions in the regulations of the German organisations (e.g., universities and graduate schools) in a qualitative approach. We, furthermore, surveyed the graduates of these organisations to find out about the practiced doctoral education in those organisations and put this into perspective with their respective regulations.

### 1.1 Doctoral education in Germany

Traditional doctoral education in Germany is based on the individual model and takes the form of a mixture of training and professional work, as most doctoral candidates are employed as junior staff members at universities and the doctoral education itself is organised rather informally [14, 21], leaving all decisions about training and guidance to supervisors and doctoral students. However, since the 1980s, there has been a debate about quality assurance in doctoral education [22, 23]. As a consequence of this debate, certain quality standards of doctoral education are being recommended, such as having a formal supervision agreement between supervisor and candidate, objective and transparent admission criteria, imparting key skills for young researchers through formal doctoral training and setting a time frame for the completion of the thesis [24].

In Germany, the first structured approaches were introduced in the 1980s by the Volkswagen Foundation (in German: Volkswagenstiftung) [24] and in the 1990s by the German Research Foundation (in German: Deutsche Forschungsgemeinschaft (DFG)) [25]. Such initiatives initiated the ongoing trend to formalize supervision, shift from a one-supervisor-cantered approach to supervisory committees, send doctoral candidates to mandatory courses that often include also interdisciplinary aspects, make admission processes more transparent, and put efforts into integrating individual doctoral studies into overarching research plans [24]. In the last two decades, many new structured approaches have emerged from the Excellence Initiative [26]. Nevertheless, after three decades, the individual model is still the dominant model of doctoral education in Germany [21]. In consequence, individual and structured approaches to doctoral education coexist in Germany.

Nevertheless, in the last few years, the percentage of structured doctoral studies has increased. The reports of the German Federal Ministry of Education and Research of 2013 and 2017 show an average increase on structured doctoral education from 8% to 23%, with a substantial difference among the academic disciplines [21]. In the natural sciences, about 33% of the doctoral candidates are enrolled in a structured programme whereas in medicine, the percentage amounts to only 14% [27]. Although Germany has the highest entry rates into advanced or post-graduate research programmes with one in twenty students being expected to enter an advanced research programme [28], structured doctoral education is still considered to be in an experimental phase [17]. Accordingly, there are various approaches to structure doctoral education in Germany with varying degrees of structure including the amount of supervision, admission criteria, formal learning opportunities as well as obligations of the candidates and their social integration into the organisation [29].

### 1.2 Aim of this paper

As described above, there are numerous recommendations from political stakeholders about the design of the structured doctoral education (e.g., from the DFG and the German Council of Science and Humanities (in German: Wissenschaftsrat (WR)), but there are no nationwide regulations in Germany. Each organisation is free to design its own doctoral education system and is responsible for conducting its doctoral training. In this paper, we aim to investigate the current state of doctoral education in Germany on three levels: the public policy level, the organisational level, and the level of individual experiences of doctoral graduates in the life sciences.

We compare the recommendations of European and German public policy stakeholders with regulations for doctoral education in individual organisations (i.e., at the faculty or programme level). Afterwards, we checked the latter against the reported experience of their graduates. With this comparison, we aim to shed light on the practices using different approaches towards doctoral education, providing the basis to assess whether and how the new and structured approaches have affected the process of doctoral education. We focus on faculties and graduates in the life sciences (medicine and bio-sciences) because the bio-sciences have the highest rates of doctoral candidates in structured programmes in Germany, whereas they are very low in medicine [21, 27]. Even though students, supervisors and researchers from these disciplines often work together in larger multidisciplinary contexts, we should be able to extract potential discipline-related differences according to the underlying undergraduate qualification, which are reflected in the doctoral degree (e.g., Doctor of Medicine or PhD or equivalent).

## 2 Methods

Different methods were used to address the three levels of interest: the political recommendations regarding doctoral education (cf. 2.1), the organisational arrangements and regulations of doctoral education (cf. 2.2) and how the practice was experienced by the graduates (cf. 2.3), as well as their comparison (cf. 2.4).

The Study is approved by the Ethics Committee of the Hospital of the Ludwig-Maximilians-Universität Munich.

### 2.1 Literature review–Political recommendations

An internet search was performed on recommendations for doctoral education released by European and German scientific and/or political stakeholder institutions. We searched on the websites of relevant institutions: European Commission, General Faculties Day (in German: Allgemeiner Fakultätentag), German University Union (in German: Deutscher Hochschulverband), DFG, German Rectors´ Conference (in German: Hochschulrektorenkonferenz), Presidents of the European Rectors´ Conference, European Science Foundation, European University Association, THESIS e.V. and WR. We used the search terms ‘doctoral training’, ‘doctoral education’, ‘doctoral studies’ (including their German equivalents). From the sources retrieved, we identified aspects that were proposed as reforms or good practices of doctoral education. Then, we solely focused on those aspects that we were able to further examine at the organisational level and at the student experience level.

### 2.2 Document analyses and structured interviews–Regulations at the organisational level

To identify regulations at the organisational level that our survey respondents were subjected to during their doctoral studies, we analysed the structured doctoral programmes or graduate schools in which they were enrolled (94 different doctoral programmes and graduate schools in total) and the doctoral regulations of their respective faculties or departments (15 in total). All of the documents describing these regulations were obtained from the official websites of the doctoral programmes, graduate schools, faculties, and departments. All documents and website texts were included that were titled as “programme description”, “doctoral regulations”, or in a similar way. The analysis of the collected documents was based on a theory-driven approach applying qualitative content analysis [30] and using qualitative data analysis. For this purpose, a category system was developed, and two coders were trained to code the data. Of the total data material, 20% was coded by both coders with 95.25% consensus (Holsti Index 0.95) and a Cohens-Kappa or K_α_ [31] of 0.95.

To confirm whether we extracted and interpreted the information gathered in the document analysis properly, we contacted representatives of the doctoral programmes. We sent them a short summary of the results of our analysis on their programme and they could either confirm or contradict the collected information on their programme in an individualised questionnaire so that we could adjust the gathered information. In order to increase participation in the study, representatives of the doctoral programmes were asked if they preferred to answer the questionnaire in writing, or in a telephone interview together with a member of the research team. 54 of the 94 questionnaires were answered by program coordinators in a written form, 15 were answered in a structured telephone interview that followed the questionnaire. As some doctoral programmes were offered for a limited amount of time and no longer existed at the time of our research, we consequently were not able to receive an answer to our questionnaire from those programmes. Some representatives did not respond to our questionnaires, even though we approached them several times. The information from the 54 completed questionnaires was integrated in the data for document analysis. Again, two coders worked on this step and an intercoder reliability test on 20% of the material was conducted, resulting in a Holsti-Index of 0.97 and a K_α_ of 0.94.

### 2.3 Multi-cohort survey–Reported doctoral education of doctoral graduates

From 2014 to 2016 –each up to one year after their respective doctoral graduation—three cohorts of doctoral graduates in the life sciences were contacted via their former examination offices to participate in an online survey, using a standardised questionnaire. The respondents were graduates (between April 2013 and March 2016) from 15 different German universities of the three federal states of Bavaria, North Rhine-Westphalia, and Saxony, and their respective medical or bio-science faculties or departments. We selected three structurally representative German states (Bavaria, North Rhine-Westphalia and Saxony) and asked all universities in these states that had both a medical school and a bio-science department or faculty for participation in our study. Of a total of 30 departments in 15 universities, 19 departments/faculties followed our call and took part in our study. The following universities participated in our study: RWTH Aachen, Ruhr-Universität Bochum, Rheinische Friedrich-Wilhelms-Universität Bonn, Technische Universität Dresden, Universität Duisburg/Essen, Heinrich-Heine-Universität Düsseldorf, Universität zu Köln. Ludwig-Maximilians-Universität München, Friedrich-Alexander-Universität Erlangen-Nürnberg, Universität Regensburg, TU München, Universität Witten/Herdecke, Julius-Maximilians-Universität Würzburg.

Within the framework of this ‘project’, we developed a standardised questionnaire that was sent out to doctoral graduates of the participating universities that captured relevant aspects of doctoral training. These included doctoral graduates’ studies prior to the doctoral training, the general framework of the doctoral training (discipline, pursued doctoral degree, specialisation, etc.), the structure of the doctoral training (e.g., membership in a doctoral programme, admission, supervision, formal learning activities, integration into a work group and the scientific community), the results of the doctoral training (grades, duration, number of publications, satisfaction with the outcome, etc.) and the first employment after graduation. The single items and scales used in the questionnaire were based on existing literature and adapted for our specific purpose and study population. Items and scales relevant to this paper are described below.

Before starting the fieldwork, we conducted a pre-test. 52 persons from the Ludwig-Maximilians-University in Munich completed the pre-test questionnaire. In general, the pre-test yielded good results particularly concerning the multi-item scales. After minor adjustments (e.g., in the wording and order of questions), the actual questionnaire was sent to the respondents via the medical or bio-science faculties of their alma mater.

Of the respondents, 42.5% (n = 764) had a previous degree (master’s or equivalent) in natural science, 44.3% (n = 796) were graduates of medicine, and 8.3% (n = 149) had graduated in dentistry. 3.1% (n = 56) and 1.7% (n = 31), respectively, held a social sciences/humanities or health sciences degree. Medical graduates and dentists were merged into one category *medicine* (*n* = 945). The group defined here as bio-science mostly has a background in biology or a biology-related field. The response rate is about 27% resulting in a sample of 1,796 valid cases. However, there are several reasons why we decided to exclude respondents with a social sciences/humanities and health sciences background beyond medicine. First, we did not collect data on these disciplines systematically. The cases in our sample are those students who for some reason or another achieved their doctorate in a medical or bio-science department. They, therefore, might not be suitable cases to represent those disciplines. Second, their sample size is quite small, 3.1% and 1.7% for social sciences and health sciences, respectively. When taking into account that only some of those respondents took part in a structured programme, the number of cases is too small for statistical analysis, in particular for multiple correspondence analysis.

The sample size, thus, is 1,712. In the surveyed sample, 349 respondents stated that they had been enrolled in structured doctoral studies across 94 doctoral programmes or graduate schools. Furthermore, 1306 respondents reported to have been involved in a traditional form of individual doctoral education. Lastly, 54 respondents did not state whether they pursued their doctorate in a structured programme or individually.

The following variables are central for the present paper:

We asked whether the respondents were enrolled in a structured doctoral programme or not.

To capture the admission criteria that the respondents experienced, we asked: “How were you accepted into your PhD program? If you pursued the individual doctorate path: how did you get your position as a PhD candidate?” (Please check all options applicable to you). The possible options were: ‘Application to a public tender’, ‘interview with an academic supervisor’, ‘interview with a panel of several professors’, ‘assessment centre’, ‘motivational letter’, ‘abstract of thesis (Master’s, Diploma, Exam)’, ‘exposé (of doctoral thesis)’, ‘contacted the employer/professor independently’, ‘with the help of colleagues or superiors’, ‘with the help of other personal contacts’, ‘I didn’t have to go through a selection procedure’.

Formal admission to doctoral education is defined here as a process where the candidates have to undergo a (competitive) selection procedure, where they must conform to certain standards. The first seven options (including exposé) are classified as formal criteria. In case our respondents stated to have obtained their doctoral position or their enrolment with help from superiors, colleagues or peers, an independent contact with a supervisor or with no selection procedures at all, they were categorised as “informal admission”. Since the respondents could select several of the options provided in the questionnaire, we created a variable with four categories from their answers: ‘formal admission’ when the respondents stated at least one of the formal procedures, ‘informal admission’ when they checked any of the informal procedures, ‘no formal criteria’ when they did not check any of the formal procedures and finally ‘no informal admission’ when the respondents did not go through any of the informal categories.

The duration of the doctoral training was calculated from the point when the respondents started to work on their doctorate (including orientation and preparation stage) until formal completion of their doctoral training (the date they received their doctoral certificate).

Concerning the supervision during the doctoral training, respondents were asked if they had signed a supervisory agreement. If they had signed one, we asked in addition, which of the following aspects were part of the agreement: ‘topic of doctoral thesis’, ‘number of supervisors’, ‘supervisors’ rights and obligations‘, ‘frequency of consultations with supervisors’, ‘your rights and obligations as a PhD candidate’, ‘time frame of the PhD program’, ‘work plan / milestones’, ‘frequent preliminary reports’, ‘number of publications’, ‘lectures at internal colloquia and symposia’, ‘visits abroad’, ‘use of resources at the institute (labs, etc.)’.

To measure formal training, 15 items were available in total. The first seven capture those types of formal learning that are commonly part of such programmes, offered on a more or less regular basis. The respondents could state whether they attended them 1 = never, 2 = once per year, 3 = rarer, 4 = at least semi-annually, 5 = at least quarterly, 6 = at least monthly, 7 = at least weakly. The respondents were asked to rate their participation with respect to these types of formal training:

- Departmental or workgroup internal colloquia of doctoral candidates, progress reports, or similar events
- Cross-institutional colloquia of doctoral candidates, progress reports, or similar events
- Lectures/seminars on topics related to your dissertation or workgroup
- Lectures/seminars on topics unrelated to your dissertation or workgroup
- Literature clubs, journal clubs, literature seminars
- Trips with your faculty, doctoral students’ day, excursions, or retreats
- How often did you present your own findings?

Additionally, respondents were asked about their attendance on eight representative forms of formal training that are usually only attended once. Respondents were asked if they had attended such a format or not. The items were:

- Scientific writing and publishing (composing professional articles and writing the dissertation)
- Methods for presenting the results (e.g., use of media, poster design, giving lectures)
- Writing grants
- Statistics (data assessment and evaluation)
- Your subject’s research methods
- Legal basis for research (e.g., radiation protection, animal welfare, drug law, genetic engineering)
- Language course (German or English)
- Career development (e.g., career planning, management skills, project management)

### 2.4 Statistical methods–Comparison of theory and practice

To compare the regulations found in the document analysis with the responses to the multi-cohort survey we present bivariate statistics. Since most of these characteristics are measured nominally and are dichotomous, we calculate coefficient Phi.

For metric data we used T-Tests to compare respondents form a structured programme to those who pursued their doctorate individually. For ordered categorical data we applied Mann-Whitney-U-Tests.

To illustrate the relationship of various structuring characteristics of the doctoral education we carried out a Multiple Correspondence Analysis (MCA) on our data. This exploratory, multivariate method is used to display columns and rows of a so-called indicator matrix that are comprised of several categorical variables [32, 33]. Based on this matrix, MCA is useful to structure categorical (nominal as well as ordinal) data [34]. It is a method to “describe, explore, summarize, and visualize information contained within a data table of N individuals described by Q categorical variables” [35]. In an indicator matrix, there is one row for each unit of analysis (i.e., respondents in our case) and one column for each category of each variable. A cell contains the value ‘0’ if the category was not chosen by the respondent and ‘1’ if it was chosen. Besides describing the relationship between variables as in related methods such as principal component analysis (PCA), the major advantage of using MCA is to investigate the relationship between single categories of variables numerically and visually [35]. To describe how far the characteristics of doctoral education corresponded to one another, we depicted the categories of the variables in a two-dimensional Cartesian coordinate system. Each category of each variable is represented by a symbol in the system. The closer two categories are located to each other, the more often they were selected by the same respondents, controlling for other variables in the model. Since the MCA is a multivariate technique, all variables are integrated in the calculation of the respective positions in the two-dimensional space. By the location of the variables’ categories, we can describe the relationships between them much more clearly than by the investigation of bi-variate cross-tables. For example, cross-tabulating five categorical variables would result in ten cross-tables, which is much more confusing than a single graph depicting the same variables and their categories.

Since an MCA can be seen as a PCA for categorical variables [35], the visualisation in the coordinate system is based on centred and standardised data and the units on the axis can be compared to standard deviations. Furthermore, we present the eigenvalue (λ) of each dimension. λ can be interpreted as the mean of squared correlation ratios of the respective dimension and the variables and is, thus, comparable to the concept of explained variance in a PCA.

When the data submitted to an MCA are ordered categorical variables, such as a multi-item Likert-type-scale, the categories are usually ordered as u-shaped or inverted u-shaped trajectory along the y-axis. This so-called horse-shoe effect is a methodological artefact [33] that is created by the ‘ordinality’ of the data. Along the first dimension, the categories of the variables are ordered in ascending or descending order and the second dimension separates the more common middle categories (i.e., usually the categories ‘2’, ‘3’ and ‘4’ on a 5-point-scale) from the less common extreme categories (e.g., ‘1’ and ‘5’). This second dimension is usually not interpreted, and focus is put on the first dimension that captures the substantial variation in the data.

There are several reasons for the application of the MCA. First, this paper is of exploratory nature and so is the MCA. It allows to display a vast array of categorical data in a condensed way. Tying in with this argument the graphical display of several contingency tables in one graph facilitates the presentation of complex categorical data including several variables and produces a more convenient illustration than comparing several contingency tables ‘manually’. Finally, we chose the MCA because it has no requirements concerning the distribution of the data. In a complex dataset including variables with varying categories that may be in a multiple relation to one another a flexible tool like the MCA is the appropriate choice.

## 3 Results

In each chapter of the result section, the three levels of interest, i.e., first, stakeholder recommendations, second, organisational regulations and third, the reported practices are described and compared to the organisations’ regulations.

### 3.1 General recommendations and admission to doctoral education

Derived from our introductory background, Table 1 depicts the general recommendation of structured doctoral education along with two areas of structure described above: Transparent and accountable admission to doctoral education and limiting the duration of doctoral studies.

**Table 1:** General recommendations on doctoral education.

General recommendations on doctoral education	Organisations/associations/foundations supporting these recommendations
**Structuring doctoral education**	*EUA 2010*, *ESF 2011*, *WR 1995*, *WR 2002* [5, 7, 36, 37]
**Establishing institutional structures for doctoral education (e.g., graduate schools)**	*ERC 2015*, *EUA 2010*, *WR 2002* [5, 7, 38]
**Transparent and accountable admission to doctoral education**	*EC 2005*, *EUA 2010*, *WR 2002* [5, 7, 39]
**Limiting the duration of doctoral studies**	*EUA 2010*, *WR 2002* [5, 7]

EC = European Commission; ERC = Presidents of the European Rectors´ Conferences; ESF = European Science Foundation; EUA = European University Association; WR: Wissenschaftsrat (German Council of Science and Humanities).

#### 3.1.1 Structure of doctoral education

Structured doctoral education and the corresponding organisational structures are recommended, e.g., by the *European University Association* [7] and the *German Council of Sciences and Humanities *[5, 34].

In all of the 15 examined locations, so-called doctoral programmes or graduate schools have been established. Six of the 15 locations have entrenched central organisational structures for doctoral education as umbrella organisations for doctoral education. Umbrella organisations are primarily formal administrative units that include all available graduate programmes, irrespective of their subfield and offer optional courses for PhD candidates [40]. Umbrella organisations typically offer optional courses, mostly for developing transferable skills, and arrange events where doctoral candidates can meet and network each other [41]. They appear to have less impact on the daily research training for doctoral candidates than doctoral programmes [40, 42]. Such doctoral programmes are typically organised along a thematic line and classically offer courses that are more specific for their respective discipline or research topic than those of their umbrella organisations [40–42].

We asked our respondents in which discipline they received their degree that qualified them for doctoral studies and whether their doctoral education was individually organised or part of a structured doctoral programme. In total, 22.8% reported a structured doctoral education and 77.2% pursued their doctorate in the individual model, with a significant difference at the disciplinary level: structured doctoral education is much more prevalent in the bio-sciences (38.3%) than in medicine (7.9%). Medical doctoral students in Germany mostly conduct their doctoral studies parallel to their regular studies, i.e., before completing their final medical exam. In medicine, a structured doctorate is perceived to cost more time and effort than individual doctoral studies. This issue is described by *Pfeiffer*, *Dimitriadis*, *Holzer*, *Reincke*, *& Fischer* [43] who found that medical candidates in a structured programme have a higher intrinsic motivation for research and, therefore, are willing to accept the additional time and effort required by structured programmes. Furthermore, most medical students want to pursue a career as practitioner and they rather have an extrinsic motivation for the doctorate. Hence, there is a lack of young medical researchers in Germany [44]. The high extrinsic and low intrinsic motivation may explain why only 7.9% of our medical graduates were enrolled in structured doctoral studies, whereas 38.3% of the bio-science respondents graduated from a structured programme.

#### 3.1.2 Admission to doctoral education

For the development of a transparent admission process to doctoral education, the *European Commission *[39] proposed an internationally accepted admission system. While in Germany most structured doctoral programmes and graduate schools have defined admission criteria, the admission process to individual doctoral education is often not transparent. Table 2 depicts to what extent graduates from structured an individual models were subjected to formal and informal admission procedures. Having gone through any formal admission procedure is more common in structured programmes while informal means were more frequently used by respondents from the individual model.

**Table 2:** Formal and informal admission.

	individual	structured	total	N
no formal admission	32.2%	15.9%	29.1%	472
formal admission	67.4%	84.1%	70.9%	1149
total	100.0%	100.0%	100.0%	1621
no informal admission	42.0%	65.5%	46.9%	761
informal admission	58.0%	34.5%	53.1%	860
total	100.0%	100.0%	100.0%	1621

Column percentages; Formal admission: Φ = 0.149; P < 0.001; Informal admission: Φ = -0.191; p < 0.001.

We found a variety of formal admission criteria in the document analysis. A formalised written application to a structured programme or graduate school was found in 54.2% of the cases, a committee interview in 34.9% and a required motivational letter in 21.7%.

Generally, formal admission criteria were reported slightly more often by graduates of structured doctoral education. Informal admission criteria, such as independently contacting supervisor, help from peer, or no selection procedures at all, were more frequently reported by graduates of individual doctoral education.

Using the common procedures ‘admission committee’ and ‘formal application’ as examples, we compared the respondents´ statements on organisational regulations of their respective structured programme. The analyses revealed some interesting discrepancies: When doctoral regulations of structured doctoral programmes or schools included an admission committee interview, 42.9% of their graduates did not report if they participated in this procedure (see Table 3). In contrast, of those programmes that did not include an admission committee interview in their regulations, 16.7% of the respondents reported to have undergone such an interview nonetheless. Similarly, a formal application to a doctoral programme was not reported by 50.0% (Table 4), even if the regulations of their doctoral programme contained that admission procedure. A formal application is almost as likely in individual doctoral education as it is in structured programmes as indicated by the low value of Phi, which would be only significant at the 10% level.

**Table 3:** Admission via ‘Admission Committee interview’ as stated in doctoral programmes regulations and the reported practices by their graduates.

	Reported by respondents	Not reported by respondents	N
Admission committee contained in programme regulations	42.9%	57.1%	33
Admission committee **not** contained in programme regulations	16.7%	83.3%	117

Number of programmes = 94; Φ = .279; p = 0.007.

**Table 4:** Admission via ‘Formal Application’ as stated in doctoral programmes regulations and the reported practices by their graduates.

	Reported by respondents	Not reported by respondents	N
Formal application contained in programme regulations	50.0%	50.0%	54
Formal application **not** contained in programme regulations	31.3%	68.8%	96

Number of programmes = 94; Φ = .191; p = 0.064.

As a caveat, we cannot firmly exclude that respondents did not remember the admission process correctly after about four or five years. Also, we tried to find more admission criteria in the doctoral regulations, like assessment centres. However, other formal admission procedures were rarely reported by the respondents.

If research is financed by third party funding, most German sponsors (such as the DFG) expect to fill doctoral positions in a competitive process. Unfortunately, whereas most graduate programmes or schools have stated that they have formal and competitive admission, many of their graduates reported otherwise. This may indicate that recruitment strategies of doctoral candidates often have not changed to the extent expected on the basis of their official regulations.

#### 3.1.3 Duration of doctoral studies

The suggestions resulting from our qualitative document analysis concur with the recommendations from several institutions: the duration of the doctoral studies should be reduced. Nearly all organisations propose a duration of exactly three years, or less common, three to four years for completing a PhD or equivalent degree. Structured medical doctoral education usually has no recommended time period.

**Fig 1:**
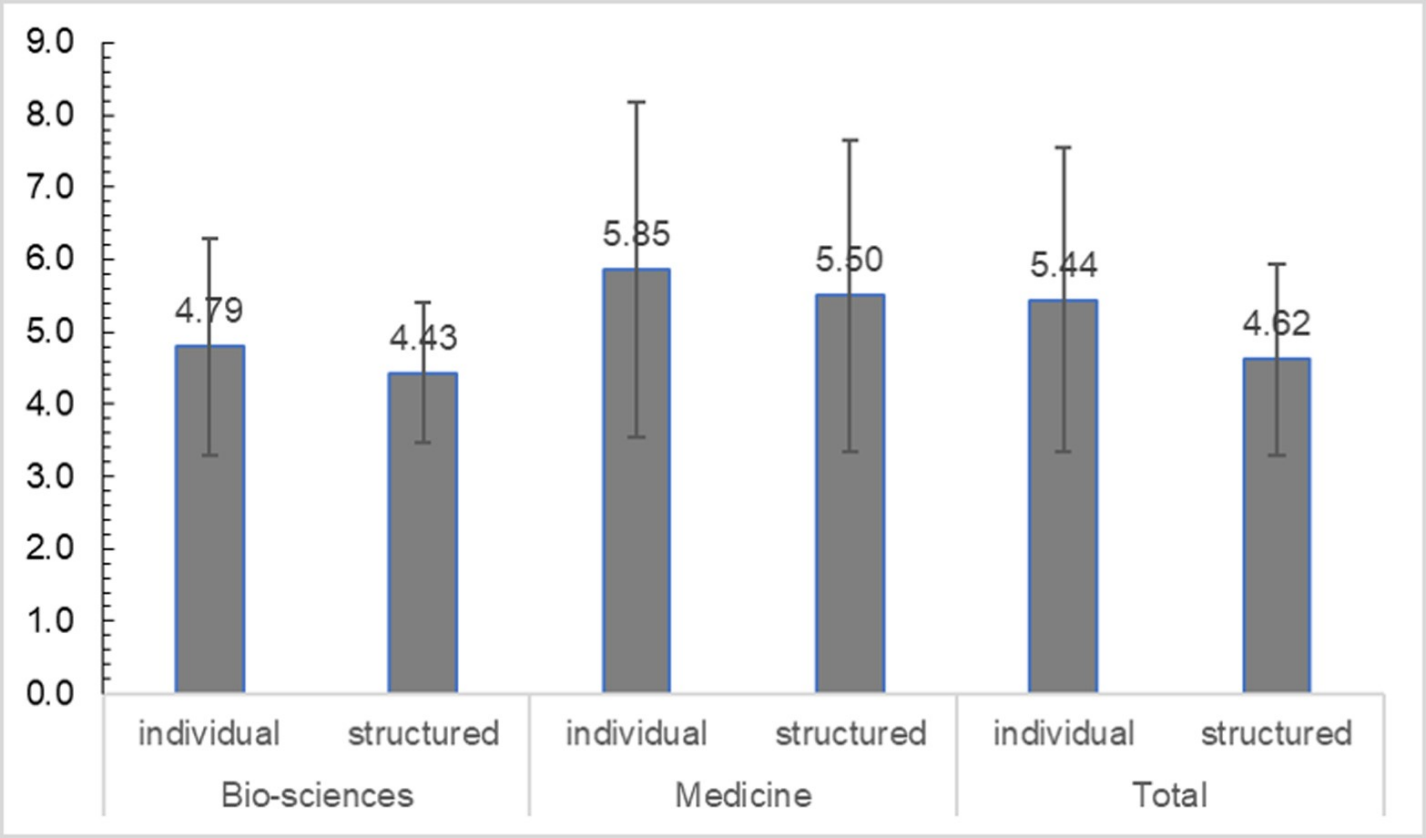
Mean duration of respondents’ doctoral education in years (from start of work to graduation). ^a^ T = 3.69, p < 0.001; ^b^ T = 1.13, p = 0.263; ^c^ T = 7.14, p < 0.001; N = 1522.

We asked our respondents when they started to work on their doctorate and when they were awarded their doctoral degree. The mean and standard deviation of the resulting period is shown in Fig 1. As it would be presumed, structured doctoral education—compared to individual doctoral education—goes along with a shorter period from starting to work on the dissertation to receiving the degree. However, if the numbers are considered by discipline, there is only a slight advantage in the bio-sciences concerning the duration of the doctoral education in structured programmes. This difference in the bio-sciences accounts for the overall difference between the structured and individual models. However, in absolute terms, the periods of time exceed the three to four years that were predefined by the organisations and institutions in any case.

It may come as a surprise that the duration of medical doctoral education is longer than that of science graduates, since in the German context, the latter usually need to put much more work in their thesis [45]. In Germany, however, most candidates from medicine start to work on their doctorate during regular studies, yet they can only finish their doctoral education afterwards, e.g., during the residency or later. Thus, the way the duration of doctoral education was operationalised in our survey cannot be taken as a valid parameter for the time or effort spent in this population.

### 3.2 Supervision of doctoral candidates

Supervision is a central topic when considering professionalisation of doctoral education and young researchers’ development. The traditional master-apprenticeship model is prone to creating one-sided dependencies between supervisor and doctoral student, possibly resulting in a disadvantage for the ‘apprentice’ [46]. More structure in the context of supervision means that the responsibility for the education of the doctoral candidates shifts from the shoulders of one person–usually a professor–to the shoulders of the organisation, as described by *Kehm *[9]. Similarly, a formal supervision agreement is expected by the funding schemes for doctoral training programmes, such as research training groups that are funded by the DFG. Other structuring activities related to supervisors (see Table 5) are associated with a more directive style of supervision [47].

**Table 5:** Recommendations on supervision in doctoral education.

Recommendations on *supervision* in doctoral education		Organisations/associations/foundations supporting these recommendations
**Supervision agreement and its contents**	Supporting supervision agreement	*AFT/DHV 2013*, *DFG 2010*, *DFG 2014*, *EC 2005*, *EUA 2010*, *ESF 2011*, *HRK 2013*, *WR 2011* [6, 7, 13, 25, 36, 39, 48, 49]
	Rights and duties of supervisors	*DFG 2010*, *DFG 2014*, *WR 2011* [6, 25, 49]
	Rights and duties of doctoral students	*DFG 2010*, *WR 2002*, *WR 2011* [5, 6, 25]
	Regular meetings between supervisor and doctoral student	*DFG 2010*, *EC 2005*, *THE/DHV 2009*, *WR 2011* [6, 25, 39]
	Determine milestones for doctoral students´ doctoral studies	*DFG 2014*, *THE/DHV 2013* [48, 49]

AFT/DHV = Allgemeiner Fakultätentag / Deutscher Hochschulverband (General Faculties Day / German University Union); DFG = Deutsche Forschungsgemeinschaft (German Research Foundation); EC = European Commission; EUA = European University Association; HRK = Hochschulrektorenkonferenz (German Rectors´ Conference); THE/DHV = THESIS e.V. / Deutscher Hochschulverband (German University Union): WR: Wissenschaftsrat (German Council of Science and Humanities).

Our respondents were asked if they had signed a written agreement with their organisation on their supervision and related aspects that structured their doctoral studies (Table 6).

**Table 6:** Supervision agreement and its contents.

	individual	structured	total	Phi
Supervision agreement	53.4%	63.7%	55.5%	-0.083**
Topic of thesis	47.4%	47.6%	47.4%	0.001
Number of supervisors	24.6%	42.2%	28.4%	0.164***
Rights and duties of supervisors	27.6%	28.1%	27.7%	0.005
Number of supervisory meetings	3.8%	29.8%	9.3%	0.365***
Rights and duties of candidate	26.8%	31.8%	27.9%	0.046
Time frame	19.1%	32.7%	22.0%	0.133***
Work schedule, milestones	9.7%	29.8%	14.0%	0.236***
Regular reports	6.4%	29.2%	11.2%	0.294***
Number of publications	2.8%	13.2%	5.0%	0.193***
Presentations in internal colloquia and conferences	1.3%	10.9%	3.3%	0.218***
Stays abroad	0.7%	8.9%	2.4%	0.218***
Utilization of resources of institution	7.6%	8.3%	7.7%	0.011
N =	1,306	359	1655	

Each cell depicts the percentage of ‘yes, applicable to me’ responses; ** p < 0.01; *** p < 0.001.

In general, a supervision agreement was more frequently reported by respondents from structured programmes than from the individual model. Concerning the particular contents of the agreement, the picture is more versatile. While the ‘topic of the thesis’ or the ‘rights and duties of supervisors’ are equally widespread in both models, issues such as ‘work schedule, milestones’ or the ‘number of publications’ were stated substantially more often by respondents from structured programmes.

**Fig 2:**
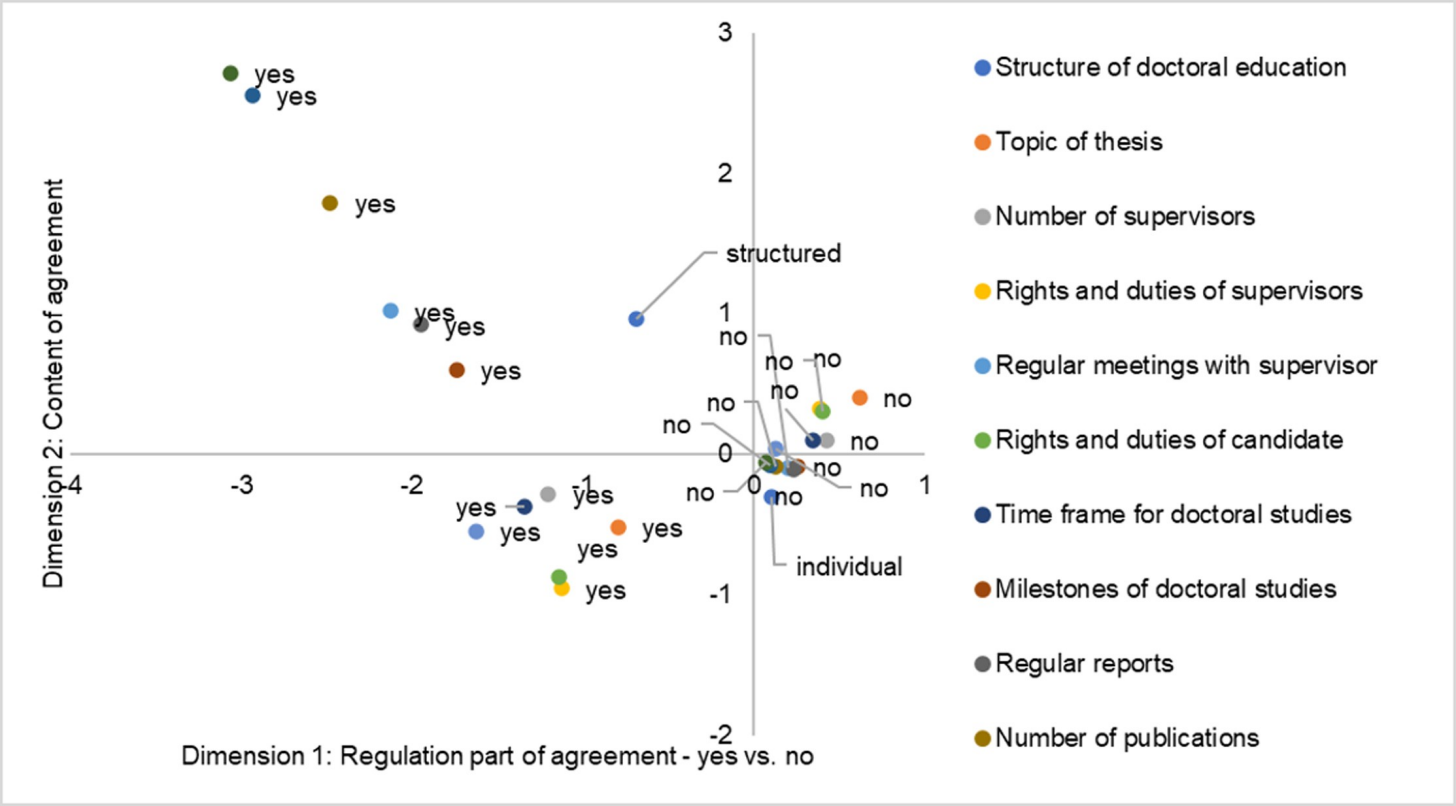
Multiple Correspondence Analysis; regulations on supervision and related aspects that might give structure to doctoral studies, as reported by our respondents; *N* = 1655.

Fig 2 mirrors the relationship between the form of the doctoral education and the regulations on supervision during the doctoral studies as reported by our respondents. Dimension 1 contrasts supervision regulations reported by our graduates of structured and individual doctoral education: In the positive part of the 1^st^ dimension all ‘no’ categories are located while the ‘yes’ categories are in the negative part. Graduates of individual doctoral education are much less likely to have any of the various regulations in their supervision agreement. While the 1^st^ dimension separates whether specific regulations were part of the agreement, the 2^nd^ dimension along the ordinate distinguishes the general content of the agreement aspects. In the positive part of the ordinate (2^nd^ dimension), we find regulations concerning temporal arrangements. In the negative part of the ordinate, the rights and duties are located. Even though the various agreements are slightly more common in structured programmes, the distance of the structured programmes to the ‘yes’ categories indicates that there is large bandwidth as to which and to what extent the regulations are implemented.

**Fig 3:**
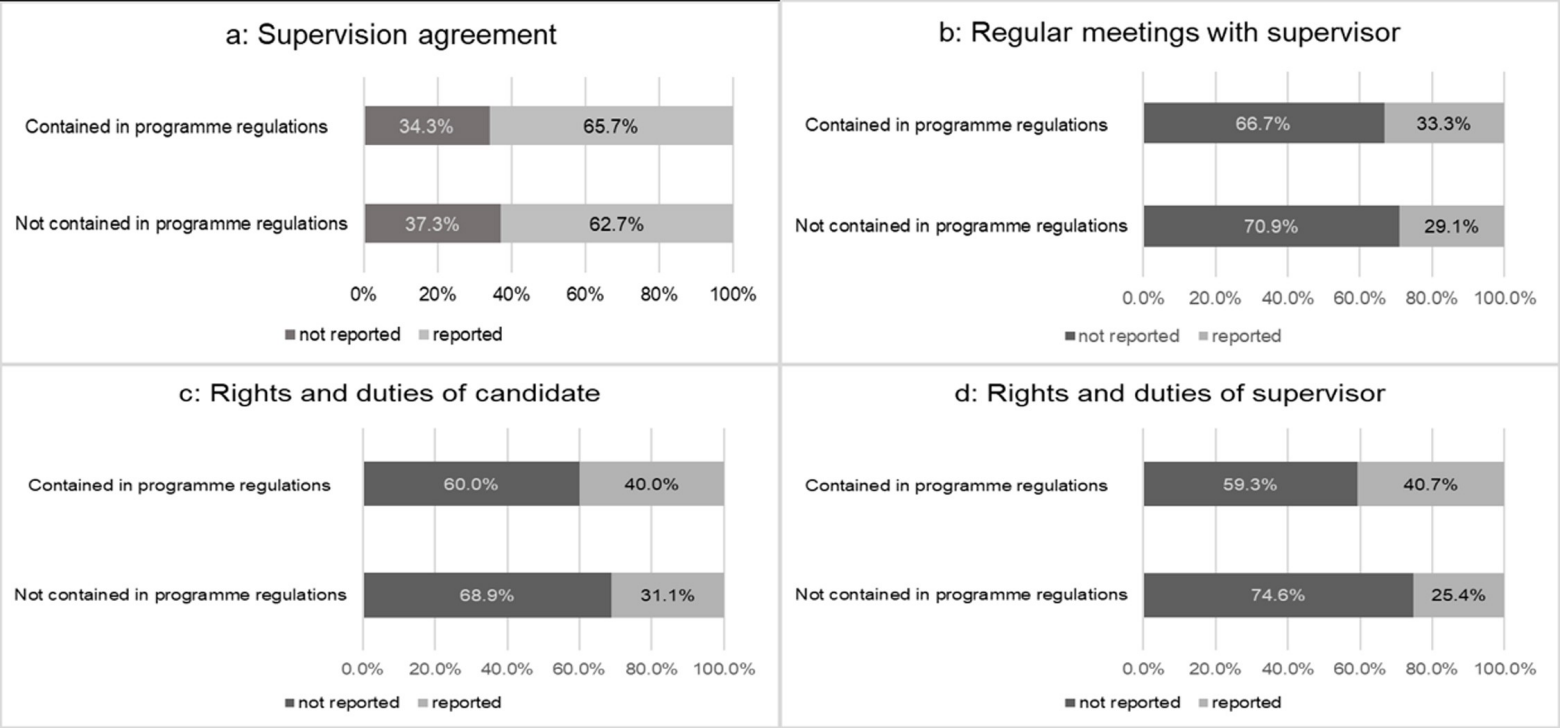
Discrepancy between doctoral regulations of structured doctoral programmes (*N* = 94) and regulatory practice as reported by graduates of structured doctoral programmes. ^a^ Φ = 0.03, *p* = 0.778; ^b^ Φ = 0.045, *p* = 0.661; ^c^ Φ = 0.078, *p* = 0.452; ^d^ Φ = 0.152, *p* = 0.140.

There were remarkable differences in the comparison of what our respondents reported and what their respective regulation contained (see Fig 3A). About one third of the graduates of structured doctoral education reported that they did not have a supervision agreement, whereas their regulations actually contained that aspect. As mentioned above, a supervision agreement is required by most funding schemes. This discrepancy is even more pronounced with respect to the three exemplary aspects as seen in in Fig 3B and 3C. About two third of the respondents did not report the experience of practice although the respective regulation was found in the programme documents. In neither case is there a significant difference between programmes that contained these regulations and those that did not. This raises the question as to why many structured programmes still deviate from this formal requirement.

### 3.3 Formal training in doctoral education

Another structuring element of doctoral education is the establishment of a formal curriculum for doctoral students (see Table 7). This is a central aspect of the career development of doctoral students as formal training should prepare them for certain tasks inside and outside academia. In particular, preparing doctoral students for non-academic careers is a very prominent and current ambition of doctoral education [2, 4, 7]. To achieve this objective, students should receive not only subject- or research-related instruction but also transferable skill training—skills that can be used in a variety of work situations [11].

**Table 7:** Recommendations on doctoral training.

Recommendations on *doctoral training* for doctoral candidates	Organisations/associations/foundations supporting these recommendations
**Implementation of curricula in doctoral education**	*ERC 2015*, *WR 2002* [5, 38]
**Credit points during doctoral studies should not be mandatory**	*EUA 2010*, *ERC 2015* [7, 38]
**Transferable skill training**	*ERC 2015*, *ESF 2009*, *EUA 2010*, *OECD 2012*, *WR 2002* [5, 7, 11, 12, 38]
**Interdisciplinary research options and feedback for doctoral students**	*ERC 2015* [38]

ERC = Presidents of the European Rectors´ Conferences; ESF = European Science Foundation; EUA = European University Association; WR: Wissenschaftsrat (German Council of Science and Humanities).

Many countries, such as Canada, France, the United States, and the United Kingdom, do not have specific strategies for implementing transferable skills in doctoral education [11]. The same can be stated for Germany, where the respective academic organisations are responsible for the formal training of their doctoral students. *Andres et al*. [1] described that there is a tendency toward a growth of graduate schools within organisations, which is linked to an increase in formal training. However, formal training is generally designed for improving doctoral students’ employability inside academia and not for developing transferable skills [11].

Table 8 provides an overview over the responses to the question asking for attendance of formal learning formats where respondents could answer with ‘yes’ or ‘no’, showing that all learning formats are more often attended in structured programmes than in the individual model.

**Table 8:** Formal learning—non-regular learning formats.

	individual	structured	total	Phi
Scientific writing and publishing	38.8	67.5	44.8	0.234***
Methods of result presentation	27.2	61.9	34.5	0.297***
Grant writing	7.7	22.8	10.8	0.197***
Statistics, data collection and analysis	47.4	53.3	48.6	0.049
Career development	14.8	54.3	23.0	0.381***
Research methods of discipline	27.3	55.3	33.2	0.243***
Legal basis for research	22.6	42.6	26.8	0.184***
Language courses	9.1	27.4	12.9	0.221***
N =	1261	332	1593	

Each cell depicts the percentage of ‘yes’ responses. *** p < 0.001.

Table 9 illustrates the attendance of learning formats offered on a regular basis such as lectures. On average, respondents from structured programmes visited all types of formats more frequently.

**Table 9:** Formal learning—regular learning formats.

	individual			structured			total		
	1st Quartile	Median	3rd Quartile	1st Quartile	Median	3rd Quartile	1st Quartile	Median	3rd Quartile
Departmental or workgroup internal colloquia of doctoral candidates, progress reports, or similar events^a^	1	4	7	6	7	7	1	5	7
Cross-institutional colloquia of doctoral candidates, progress reports or similar events^b^	1	1	4	2	5	6	1	2	5
Lectures/seminars on topics related to your dissertation or workgroup^c^	1	3	5	4	6	6	1	3	6
Lectures/seminars on topics unrelated to your dissertation or workgroup^d^	1	3	6	3	5	6	1	4	6
Literature clubs, journal clubs, literature seminars^e^	1	1	5	4	6	7	1	3	6
Trips with your faculty, doctoral students’ day, excursions or retreats^f^	1	1	2	2	2	3	1	2	3
How often did you present your own findings?^g^	2	4	5	4	4	5	3	4	5

N = 1599; Response categories: 7 'once per week'; 6 'once per month'; 5 ' every three months'; 4 ' every six months'; 3 'once a year'; 2 'less than once a year'; 1 'never'; Mann-Whitney-U-Tests: ^a^ Z = -13.71 p < 0.001; ^b^ Z = -13.78 p < 0.001; ^c^ Z = -13.38 p < 0.001; ^d^ Z = -7.12 p < 0.001; ^e^ = -13.81 p < 0.001; ^f^ Z = -12.91 p < 0.001; ^g^ Z = -7.80 p < 0.001.

**Fig 4:**
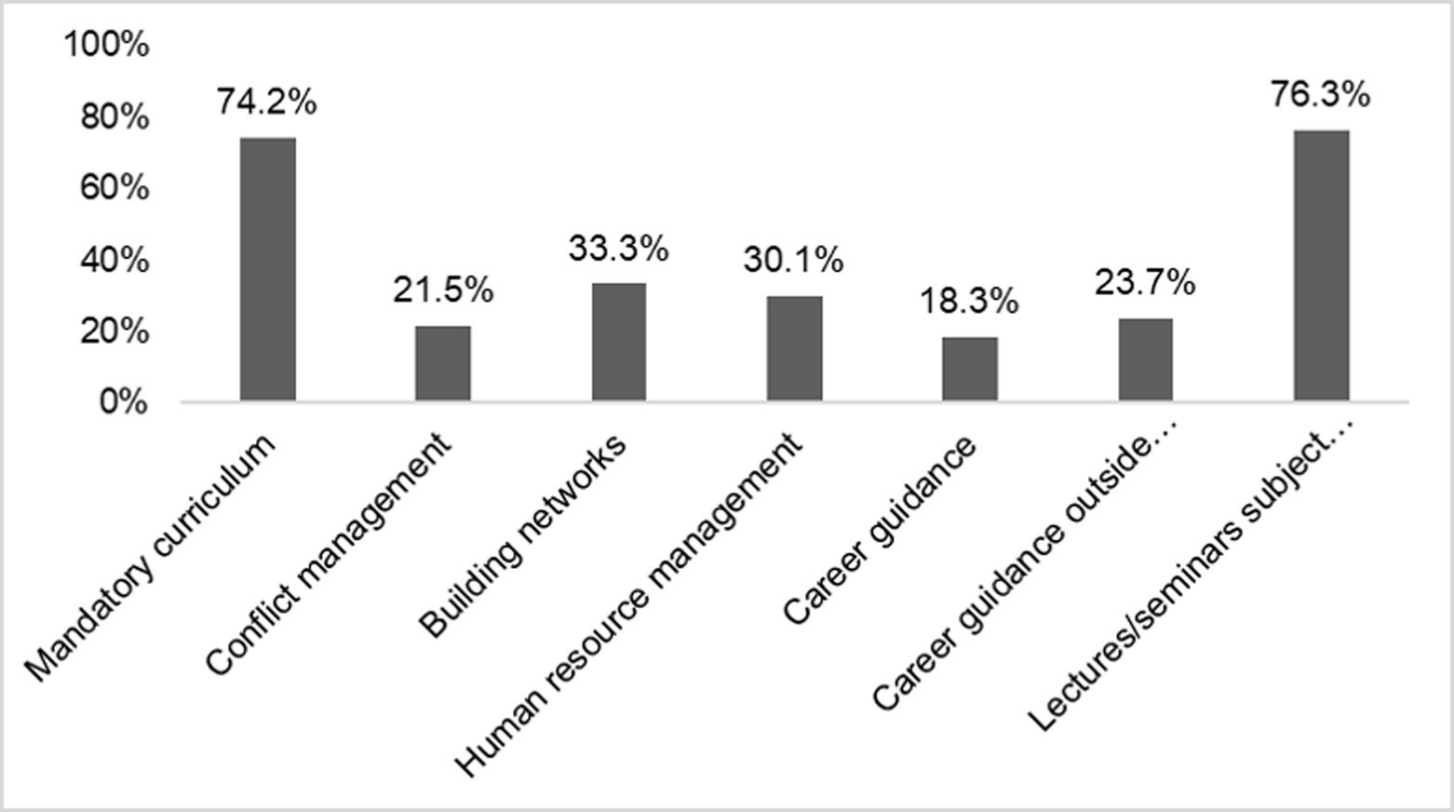
Types of courses that are offered or mandatory in doctoral regulations; *N* = 93.

As depicted in Fig 4, most of the analysed programme documents included a mandatory curriculum. This curriculum comprises a vast array of courses, lectures, and other formal learning formats, e.g., retreats or summer schools. Using five such learning formats as examples, we demonstrate that lectures and seminars with subject related contents are quite widespread whereas transferable skill training (e.g., conflict management or building networks) or career relevant learning opportunities are much scarcer.

**Fig 5:**
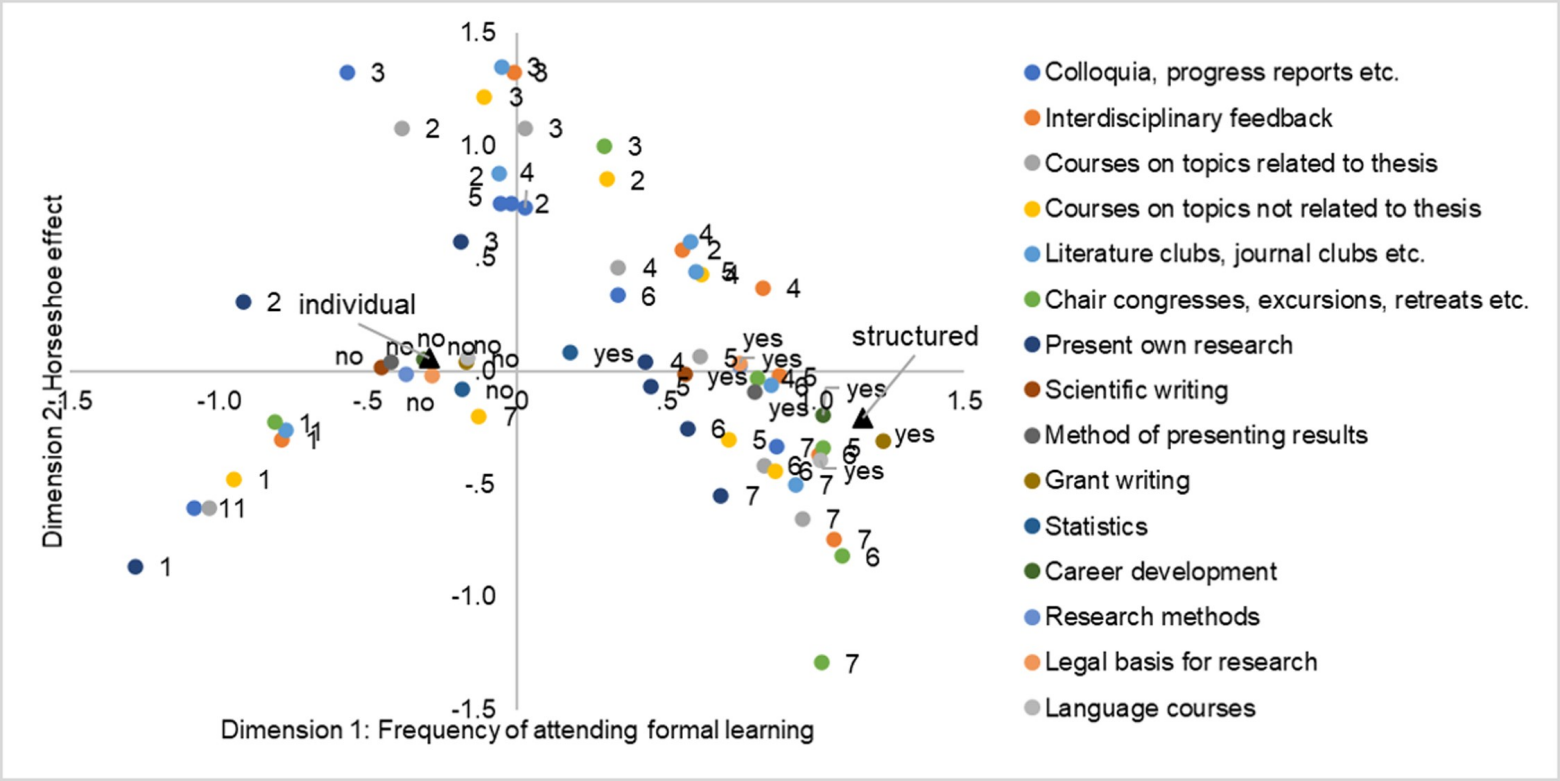
Multiple Correspondence Analysis; formal doctoral training that our respondents report to have attended during their doctoral studies; *N* = 1290; response categories of categorical items: 1 = never, 2 = once per year, 3 = rarer, 4 = at least semi-annual, 5 = at least quarterly, 6 = at least monthly, 7 = at least weakly.

Our respondents were asked which types of learning formats they had experienced during their doctoral studies. Fig 5 displays the results of the MCA including all 15 items capturing formal learning and the structured and individual model. This representation of the structuring characteristics of doctoral education offers a quite clear picture. The major contrasts are found along the first, horizontal dimension, which clearly separates structured from individual doctoral education: Higher values indicate more structure while lower values indicate less structure in terms of formal learning. The second dimension is the aforementioned methodological artefact, the so-called horse-shoe effect (see method section) and in this case separates the extreme from the moderate responses [33] of the seven-point scales used for seven of the items. Since the second dimension captures methodologically induced variation, caused by the fact that extreme responses (e.g., the categories 1 and 7) are much less common than more moderate answers, we only interpret the first dimension. The picture is quite clear, though. Formal learning formats that can be attended on a regular basis are more strongly frequented by respondents from structured programmes. Respondents with the highest frequencies of visiting these formal learning formats (i.e., selecting categories 5 to 7) are mostly those that also participated in a structured programme. The contrast between structured and individual doctoral education becomes even more pronounced when looking at the formal learning opportunities that are (usually) only visited once. All the ‘no, not attended’ answers are located close to the individual model. The ‘yes, attended’ answers are much closer to the structured model but with much more dispersion, i.e., not all programmes integrate these formats into their curriculum to the same extent.

**Fig 6:**
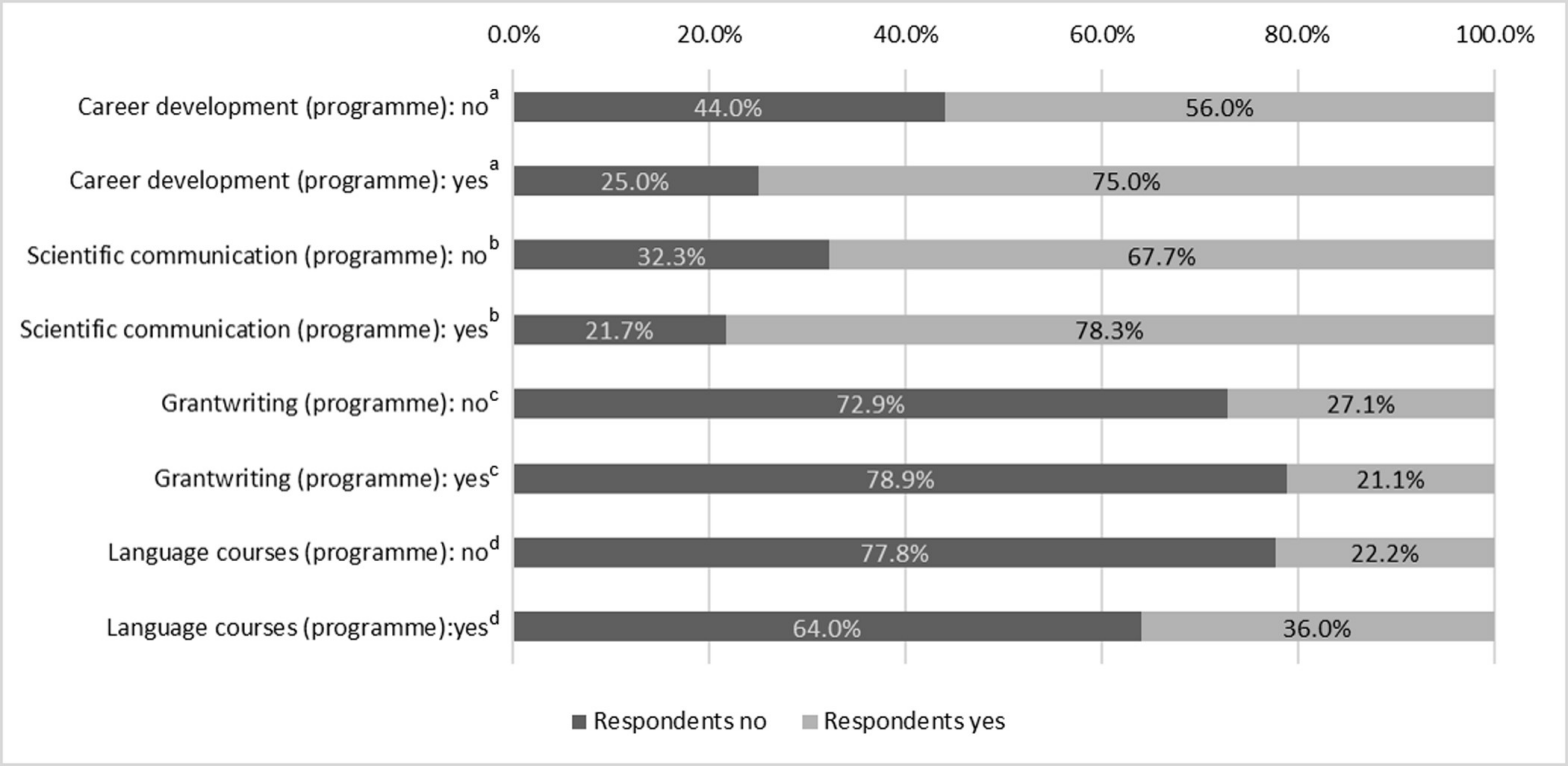
Regulations on doctoral training of structured doctoral programmes and practices reported by their graduates (*N* = 229). ^a^ Φ = 0.147, p = 0.160; ^b^ Φ = 0.116, p = 0.271; ^c^ Φ = 0.057, p = 0.591; ^d^ Φ = 0.141, p = 0.185.

Fig 6 cross-references the results from the document analysis and the answers our respondents provided on four exemplary formal learning opportunities. This example of four types of formal learning formats depicts that there is barely any difference in the responses to our survey irrespective of whether the specific course types were included in the programme documents or not. Career development courses, scientific community training as well as language courses tend to have been attended more often when the programme actually included them in its statutes, but these potential differences did not reach statistical significance. However, the data clearly demonstrates that even in cases where the respective formal learning format was specifically mentioned in the programme documents, far from all the programmes’ graduates report having participated in them.

**Fig 7:**
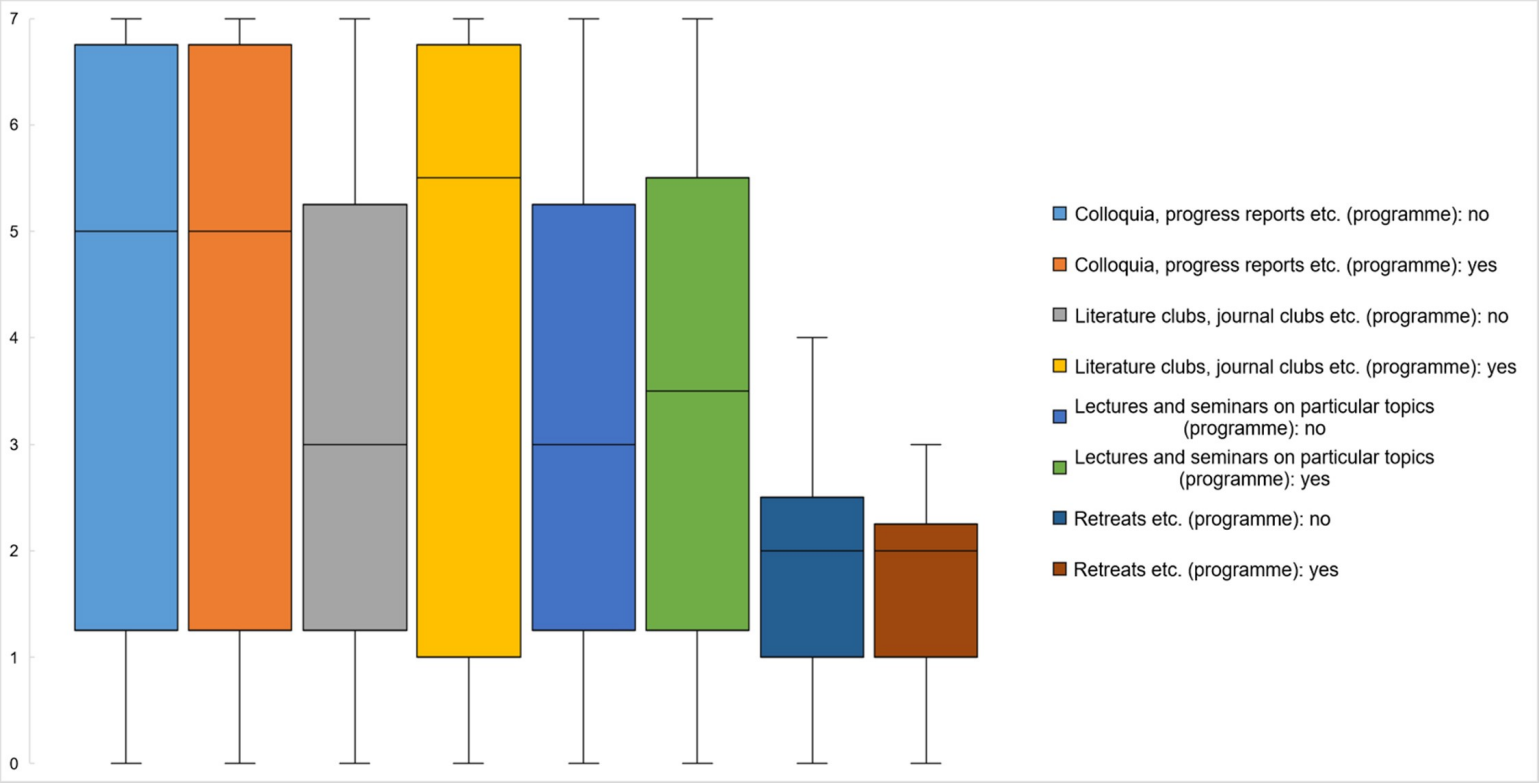
Frequency of visiting formal learning formats and programme regulations (*N* = 229).

In Fig 7, we illustrate the relationship between the results from the document analysis with the answers to our survey for the types of learning formats that are usually visited more often than only once or twice during doctoral education. In the boxplots, we compare the median frequencies of attending these courses of programmes that included them in their curriculum and those that did not. Again, there is barely any difference between programmes that included these courses in their curriculum and those that did not concerning the responses of their graduates. Only literature clubs, journal clubs, etc. were visited more often when the programme included them (U-Test: Z = -2.65; p = 0.008). However, formats such as colloquia, progress reports, the aforementioned literature or journal clubs as well as lectures are quite common and were attended on a weekly (a value of ‘7’) or monthly (a value of ‘6’) basis by many respondents. Since retreats (and similar formats) are generally much less frequent, the majority attended them annually (a value of ‘2’) to semi-annually (a value of ‘4’) if the regulations included them or to a maximum of ‘3’, ‘less than semi-annually’.

## 4 Discussion

This paper features insights into the practices of structured doctoral education in the life sciences (medicine and bio-sciences) in Germany. By comparing graduates of individual doctoral education with those of structured doctoral education models, we find that the actual differences in doctoral training are much less pronounced than expected. Our second endeavour was the comparison of organisational regulations concerning the structure of the doctoral training to the actual experiences of doctoral graduates from these programmes. Our results show that there is still a lack between aspiration of the programmes and the experienced reality.

To improve the transparency of the access to doctoral education, formal admission regulations—such as a formal application—are expected to be put into practice by funding schemes, e.g., of the DFG. A formal admission procedure to doctoral studies was observed more often in structured doctoral education than in the individual model, whereas informal criteria were more frequent in the individual model. In traditional German doctoral education, professors prefer to recruit their doctoral students from among their own students, i.e., informally. This still seems to be a dominant practice of recruitment. Surprisingly, in our analyses we found that various formal and informal admission procedures are equally implemented by the programmes whether their statutes included them or not. Our findings indicate that various forms of formal and informal admission coexist within the same programme. For example, some candidates might have applied formally while others were recommended by their professor.

Concerning the duration of the doctoral training, there is a small advantage for the structured model in the life-sciences. However, this advantage is not very pronounced and less than half a year. Furthermore, even though nearly all programme documents restricted the duration to three, in some cases up to four years, on average, the respondents needed more than four years to achieve their doctorate. An important research question for future research would be, why graduates from structured programmes almost worked as long on their doctorate as those from the individual model. Is it due to the–often only–moderate differences between the structured and the individual model? Or is it the other way around, does the higher amount of structure (e.g., more formal learning, more duties in work groups, etc.) consume time the candidates might need to work on their thesis? As an example, *Brechelmacher et al*. [50] reported that most PhD candidates feel pressurised to finish their work in three to four years, as required by most structured programmes. This might be caused by the overburden of their duties, e.g., teaching, supervising students, supporting their professor, administrative tasks, publishing and writing their own dissertation [50, 51]. In addition, doctoral students in structured programmes often have to visit additional courses as further required activities [29, 50].

Generally, we found that a supervision agreement is slightly more common in structured programmes than in the individual model. While the implementation of the rights and duties of the candidate are more often included in structured programmes, there is no difference between the individual model and structured programmes concerning the rights and duties of the supervisor. As consequence, at least concerning the candidates’ responsibilities, structured programmes offer higher transparency. In contrast, regular meetings with the supervisor are rarely part of an agreement as experienced by the respondents in the individual model whereas about thirty percent had regular meetings with their supervisor in the structured approach. Since insufficient supervision is a point of major criticism of the individual model, in that regard, structured programmes seem to be in favour. Likewise, while a work schedule and milestones can be important structuring elements. However, in the individual model only about 10 percent reported that their agreement included them while among the graduates from structured models, about 30 percent stated that a work schedule and milestones were part of their agreement.

As depicted by the MCA, all of the typical topics of the agreement are more commonly associated with structured programmes; however, there is a large dispersion of the categories. Concerning the supervision agreement and its concrete explication, the structured programmes seem to be lacking behind the recommendations of political stakeholders concerning this issue.

Contrasting the organisational regulations with the experienced practice of our respondents shows that a supervision agreement is not encountered more often even when it was included in the regulations. The same was true for the three exemplary topics of such regulations. As with admission to doctoral education, there is still a discrepancy between aspiration of the programmes, the experienced reality of their graduates as well as the recommendations of the science stakeholders. As a result, this insufficient supervision may have severe consequences. As *Berning and Falk* [52] reported a higher drop-out rate for doctoral students who lacked supervision during their dissertation phase. Accordingly, structured programmes might be able to reduce this drop-out rate of doctoral students by improved supervision [40]. Consistently, *Scaffidi and Berman* [53] have shown that mentor support is critical for the success of young academics’ doctoral studies.

Doctoral training mostly prepares students for competencies required inside academia. This agenda setting, as reported by the *OECD *[11] and as pointed out by *Andres et al*. [1], was confirmed by our findings: The formal learning formats most visited are relevant for an academic career. Furthermore, we found that training for skills that are important after receiving the doctoral degree were more likely attended by graduates of structured doctoral studies than by those of the individual model. Consequently, research should focus on labour market transitions and career trajectories after receiving the doctorate with the focus on the structuring elements of the doctoral training and their effect on this transition.

Transferable skill training courses and courses that intend to prepare for the next, more independent phase of an academic career are found more often in structured doctoral studies as shown by the associations in the MCA. However, when it comes to comparing the regulations of structured doctoral education with the reality as reported by their graduates, compliance with structuring features is again—at best—moderate.

As our results indicate, even in non-structured forms of doctoral education, there is often some “structure”. This can be due to general regulations of the university or the respective departments, in such that they require these measures irrespective of the candidates’ participation in a structured programme or not.

Although diversity in ways how a doctoral degree can be obtained is to some extent desirable as it allows supervisors and doctoral candidates to find a way that fits for them individually, the identified intransparency even within the limited context of doctoral education in German life-sciences shows a big problem for all stakeholders to take advantage of this diversity [58]. Lack of understanding of the doctoral education system has been identified as a major problem on the European level previously [58] and the discrepancy between formal regulations and actual practice we identified in this paper may be a major cause for this.

Of note, our results are biased in several ways since only successful graduates were examined: This systematically excludes the experiences of non-successful candidates. Furthermore, the survey data may be compromised by imperfect memory, in particular with respect to items addressing the early phase of doctoral studies.

As *Hauss et al*. [24] pointed out, it needs to be discussed whether the dichotomous distinction between structured and traditional forms of doctoral education is appropriate when analysing the conditions and consequences of doctoral studies. Our results add to this discussion by showing that the difference in the practices of structured and traditional forms of doctoral education is much smaller than would be expected from the underlying organisational regulations and institutional recommendations. Also, our analyses suggest that a ‘continuous approach’ towards structured doctoral education with the purely structured and the purely individual model only as the ends of this continuum would be more appropriate. Therefore, research on quality in doctoral education should focus more on actual practices—and hence the degree of implementation of structuring measures—rather than on written regulations.

Furthermore, more research is needed that investigates to what extent the structuring characteristics–be they implemented as part of a structured programme or as an integral part of doctoral education–actually exert positive effects on the outcome of the education and the graduates’ first career steps afterwards [e.g., 54, 55]. Descriptive results of the present paper not only inform about the situation in Germany but may also help to guide investigations about structuring characteristics as success factors for doctoral education in general.

Finally, in the present study, we did not address the level of individual work groups or laboratories where a particular structure might exist [56, 57]–e.g., an apprenticeship model based on the constructivist assumption that the doctoral candidates learn via interaction, by observing, imitating or following role models–again, irrespective of structured programmes, that might create a form of structured doctoral education. A deeper investigation of work groups or laboratories could add important additional information to the findings concerning practices and regulations in doctoral education presented in this paper.

## 5 Conclusion

Our results indicate that the step has been taken successfully from policy recommendations to formalization in structured programs and institutional regulations for doctoral education. However, actual implementation into practice is still an issue to be addressed. It seems unlikely that this phenomenon is specific to the life-sciences in Germany. Therefore, we recommend focussing in the next step on the professorial level that is responsible for supervision of individual doctoral candidates and work with them to reduce existing resentments and resistance. More evidence on the implementation and the actual effects of structuring elements, and practice-based examples on how this form of doctoral education is adjusted individually could be helpful. Besides the known impact of training and mentoring on supervision [58], it will take such knowledge to foster the necessary academic culture and convince professors to change their supervision strategies.
